# Does the Diabetic Foot Have a Significant Impact on Selected Psychological or Social Characteristics of Patients with Diabetes Mellitus?

**DOI:** 10.1155/2014/371938

**Published:** 2014-03-25

**Authors:** Vladimíra Fejfarová, Alexandra Jirkovská, Eva Dragomirecká, Frances Game, Robert Bém, Michal Dubský, Veronika Wosková, Marta Křížová, Jelena Skibová, Stephanie Wu

**Affiliations:** ^1^Diabetes Center, Institute for Clinical and Experimental Medicine, Vídeňská 1958, 140 21 Prague, Czech Republic; ^2^Department of Social Work, Faculty of Arts, Charles University, 116 42 Prague, Czech Republic; ^3^Diabetes Unit, Derby Hospitals NHS Foundation Trust, Derby DU22 3NE, UK; ^4^Center for Lower Extremity Ambulatory Research, Dr. William M. Scholl College of Podiatric Medicine, Rosalind Franklin University of Medicine and Science, Chicago, IL 60064, USA

## Abstract

The aim of our case-control study was to compare selected psychological and social characteristics between diabetic patients with and without the DF (controls). *Methods*. 104 patients with and 48 without DF were included into our study. Both study groups were compared in terms of selected psychosocial characteristics. *Results*. Compared to controls, patients with DF had a significantly worse quality of life in the area of health and standard of living as shown by lower physical health domain (12.7 ± 2.8 versus 14.7 ± 2.5; *P* < 0.001) and environment domain (14.1 ± 2.2 versus 15 ± 1.8; *P* < 0.01) that negatively correlated with diabetes duration (*r* = −0.061; *P* = 0.003). Patients with DF subjectively felt more depressed in contrast to controls (24.5 versus 7.3%; *P* < 0.05); however, the depressive tuning was objectively proven in higher percentage in both study groups (83.2 versus 89.6; NS). We observed a significantly lower level of achieved education (*P* < 0.01), more patients with disability pensions (*P* < 0.01), and low self-support (*P* < 0.001) in patients with the DF compared to controls. In the subgroup of patients with a previous major amputation and DF (*n* = 6), there were significantly worse outcomes as in the environment domain (*P* < 0.01), employment status, and stress readaptation (*P* < 0.01) in contrast to the main study groups. *Conclusions*. Patients with DF had a predominantly worse standard of living. In contrast to our expectations, patients with DF appeared to have good stress tolerability and mental health (with the exception of patients with previous major amputation) and did not reveal severe forms of depression or any associated consequences.

## 1. Introduction

Previous studies have shown that patients with diabetes mellitus may have a higher tendency to psychological disturbance, possibly secondary to the chronicity of the disease [1]. The most notable is the issue of depression and its possible association with diabetes [2, 3]. This could lead to the deterioration of glycemic control, subsequently increasing the risk of the development of late complications of diabetes including distal sensorimotoric neuropathy and the development of diabetic foot ulceration or acute Charcot neuroarthropathy, the diabetic foot (DF). These complications, along with the associated physical restrictions of therapy, may negatively affect quality of life and further worsen depression [4]. It has been shown that physically restrictive regimes including the necessity to offload lower limbs could result in increased psychological pressure [5]. Progression of DF may unfortunately lead to lower limb amputation in a significant proportion of patients which may cause further loss of mobility. This might not only negatively affect the psychological state of these patients worsening their quality of life [6] but could also impact their socioeconomic status.

Patients with DF can thus enter a vicious cycle—the mental stress afforded by physically limiting management strategies designed to facilitate healing of DF wounds or resolution of an active Charcot neuroarthropathy can lead to chronic stress which could potentially negatively influence patient compliance with treatment and thus outcomes [7–9]. Stress induced immune dysregulation could potentially increase the risk of serious infectious complications of foot ulceration possibly resulting in lower limb amputation [7, 8, 10].

The aim of our study was therefore to evaluate the impact of DF on daily life, particularly on the psychological and socioeconomic aspects of the patients, and compare such findings to patients with diabetes but no DF complications.

## 2. Subjects, Materials, and Methods

This was a case-control study including 104 consecutive patients with diabetic foot ulcers and/or Charcot neuroarthropathy who were treated in our foot clinic from March 2010 to March 2011 and who had no history of severe psychiatric illness (DF group, Table 1). 52% (54/104) of patients had had previous lower limb amputation (89% minor and 11% major). The control group (C group) consisted of 48 patients with diabetes matched for gender, age, type of diabetes, and glycemic control but who had no history of DF or other serious late complications of diabetes with the exception of painless peripheral sensory neuropathy (27.1% of patients). Peripheral sensory neuropathy was defined as a vibration perception threshold above 25 V [11]. Diabetic patients were consecutively included into the C group during their regular outpatient control and matched 1 : 2 to patients from the DF group. Baseline characteristics of both groups are shown in Table 1; the only significant differences included a longer duration of diabetes and a higher incidence of peripheral sensory neuropathy in the DF group.

The psychological variables assessed were quality of life, depression, pain perception, evaluation of stress impact, social support, and social characteristics.

The World Health Organization Quality of Life Assessment (WHOQOL-BREF) consists of 24 items grouped into 4 domains (physical health, psychological health, social relationships, and environment) plus 2 items of general evaluation [12, 13]. In contrast to the SF-36 instrument, the WHOQOL-BREF validity has been demonstrated to be good in all except the social domain [14]. Therefore, additional instruments to investigate the social area (i.e., social support measurement) were used in this study.

Depression was assessed using the geriatric depression scale (GDS) which includes 30 questions on the presence of depressive symptoms [15, 16]. The instrument covers four areas such as positive experiences, feelings of sadness, reduced performance/apathy, and avoidance of social contacts. The absence of depression is defined as 0 to 9 positive responses, mild depression as 10 to 19 positive responses, and severe depression (clinical depression) as 20 or more positive answers. In addition, patients rated their subjective feeling of depression and suicidal tendencies in the patient questionnaire consisting of questions on anamnestic data.

Pain was evaluated with a visual analogue scale (VAS), where 0–39 corresponded to severe pain, 40–60 to moderate pain, and 61–100 to mild or no pain [17].

To evaluate psychosocial stress, we used the social readaptation scale, scale of psychosocial load and stress, and Bortner self-evaluation scale [18, 19]. The social readaptation scale is designed to identify the impact of social consequences of active stress particularly on the degree of somatization. It consists of 43 items of various life situations that a patient might have experienced during the past 12 months. Each item has its own quantifiable weight expressed in a number of points. In the case of increased frequency of a specific situation presented during the period, points are multiplied by this frequency. Patients with no risk of somatization usually score <150 points, patients with 150–200 points might somatize (40% of cases), patients with 200–299 points develop psychosomatic disorder in 50% of cases, and patients with a score above 300 somatize in up to 80% of cases.

For the description of stressors, we used a modified questionnaire from the Stanford project of five cities [20], the scale of psychosocial load and stress, mapping two areas—the personal and professional background of each patient. This seven-point scale includes degrees of satisfaction in relation to friends, work, financial situation, health, and quality of marital or partner relationship. This scale also maps the frequency of experiences with lack of time, excessive work demands, feelings of uncertainty, and dissatisfaction at work and of considerable impatience, frustration, and sleep disorders. If the values of the 2 basic areas of the questionnaire are scored ≤10 and/or ≥21, a higher incidence of stressors is present in the patient.

The Bortner scale was used to detect psychosocial risk factors. This test is intended to search for extreme-risk behaviors and attitudes, often occurring in people at risk of some chronic somatic diseases and deteriorated mental condition. This measurement gives information about type of behavior and also serves as an indicator of level of irritability, tension, hostility, interpersonal sensitivity, experience of life events, and level of frustration. A risk value was considered as a score higher than 22 [21, 22].

For other psychosocial and socioeconomic assessments, we used a 5-item level of social support measurement. A deficit of social support was defined as a score ≥18 points [23].

Patients also completed a patient questionnaire including demographic and other anamnestic data such as level of education, employment status, self-support rate, and information about foot care.

Comparisons between the DF group and non-DF diabetic controls were performed using BMDP statistical software (Release 8.1). Descriptive data are presented as mean ± SD (after adjustment ± SE, resp.) or as percentages; differences between the two groups were determined using *t*-test or Mann Whitney test when appropriate; Kruskal-Wallis test was used for three-group testing; chi-square test in frequency tables detected discrete variables; one-way analysis of covariance (ANCOVA) and logistic regression were used for the adjustment for diabetes mellitus duration. *P* < 0.05 was considered as statistically significant.

This work was approved by the local ethical committee. Each patient signed informed consent prior to the inclusion in the study.

## 3. Results

The quality of life scores differed between the DF groups and non-DF controls in the physical health domain (*P* < 0.001) and environment domain (*P* < 0.01; Table 2) that negatively correlated with diabetes duration (*r* = −0.061; *P* = 0.003). Depression scores did not differ significantly between the DF group (12.7 ± 2.8; median 13) and control group (11.8 ± 2.2; median 11). A more detailed analysis did not show any significant difference in the incidence of various forms of depression between both study groups (Table 2). However, these data contradicted those from the subjectively described feelings of depression where patients with DF reported themselves depression significantly more frequently than controls (24.5 versus 7.3% of patients; *P* < 0.05). Suicidal tendencies did not differ significantly between the groups (5.2% in DF group versus 4.4% of controls; NS).

The VAS scale demonstrated no differences in pain perception between both study groups (DF group 62.9 ± 25.4 versus C group 69 ± 27). The distribution of severe, moderate, or mild forms of pain also did not differ significantly between the DF patients and controls—severe 24.7 versus 13.3%, moderate 23.8 versus 28.9% and mild pain 51.5 versus 57.8% of patients, respectively.

The mean scores of the social readaptation scale were significantly higher in the DF group (124 ± 112.2) compared to controls (84.5 ± 90.9; *P* < 0.05). Neither study group however showed a particular tendency to oversomatization (Table 2) nor did there appear to be an increase in the incidence of stressors based on the scale of psychological load and stress in the DF group compared to controls (32.8% versus 27.8% of patients; NS). Risk type behavior as detected by the Bortner Scale was noted in 25.3% of patients with DF compared to 17.4% of controls; this was, however, statistically nonsignificant.

Additionally, we found no significant differences in social support (deficit was reported in 16.5% of patients with DF and 14.3% of controls; NS).

Significant differences were however found in education—patients with DF had more basic education in contrast to control subjects (Table 3). In terms of employment status, patients from the DF group received more disability pensions and fewer of them were in full-time employment or conducted a business.

The largest difference found between the 2 groups was in the lack of self-support in the DF patients compared to controls (*P* < 0.001; Table 3). DF care was mostly provided by the patient themselves (49.5% of patients) rather than by their partners (34.3%). DF care was provided by other family members only in 7.1% of the cases and by home-care nurses in 9.1% of cases. Except for environment domain, all above mentioned data were statistically significant even after adjustment for diabetes mellitus duration.

In subgroup analysis, there were no differences found between control patients with and without sensory peripheral diabetic neuropathy in any of the measures (data not shown). Within the DF group, patients with a previous major amputation differed significantly only in one parameter of QoL (the environment domain—11.7 versus 14.2 and 15.0; *P* < 0.01) and in employment status (percentage of patients on disability pensions—100 versus 41.2 and 6.2%; *P* < 0.01) in contrast to other patients with DF and diabetic controls without DF. Based on the stress readaptation scale, amputated patients had significantly worse stress readaptation in contrast to other patients with DF and diabetic controls (187 versus 119 and 84.5; *P* < 0.01).

## 4. Discussion

In this study, we aimed to examine in detail the impact of DF on quality of life, chronic psychological stress, especially in terms of social issues, and the socioeconomic sphere. Social problems and the impact of stress together with readaptation to chronic stress have not, thus far, been studied in patients with DF.

In this study, patients with DF scored significantly lower in a domain of the QoL questionnaire relating to the environment relating negatively to diabetes duration. This domain involves the patient's finances, quality of living, and the environment that closely correlates with standard of living. In the subgroup of DF patients who had had an amputation, these scores were even lower. Lower standards of living in patients with DF are a reflection of a higher percentage of patients on disability pensions, fewer patients in employment, and a lower level of education. The association of lower social economic level with a higher incidence of late diabetic complications [24] has not been frequently described, but it is possible that once established, late complications, particularly complications affecting the foot, could affect this area of life, by limiting social contacts [19, 25] causing isolation and imposing unemployment from limited mobility. This may subsequently result in financial problems and hardship [6].

Unsatisfactory social conditions in patients with DF are also supported by the finding of significantly reduced self-support reported by our patients with DF. This again could be a consequence of decreased mobility, including the necessity for offloading, as well as lower self-care generally described in diabetic population [26]. Although self-support and consequently self-care was significantly reduced, almost half of the patients according to the questionnaires were taking care of their feet by themselves. This may reflect the fact that, in our country (where there are insufficiently developed home-care services systems), patients usually take care of their own health and utilize health services infrequently.

Improvement of physical capacity (e.g., by exercise with adequate offloading), and in particular of self-support/self-care, could perhaps improve the psychological comfort of patients and in turn improve patient adherence to DF therapy leading potentially to better wound healing [7]. However, enhancement of their knowledge may not necessarily significantly improve patients DF self-care, although the introduction of motivational-interventional interviewing techniques may be helpful according to some experts [26].

In contrast to the control group of patients with diabetes but no DF, poor social conditions together with the chronicity of DF complications could contribute to the alteration of quality of life in the area of physical health. Other factors potentially negatively influencing a health status include the number of physical restrictions imposed upon patients as part of their management and the higher number of comorbidities associated with DF. Similar changes in quality of life shown in this study have also been described in other published studies [27–31].

Depression has been shown to be associated with an increased risk of the development of DF possibly due to its negative effects on patient compliance and glycemic control and consequently on wound healing. Patients with DF did not have a significantly higher prevalence of severe depression compared to diabetic controls (1% versus 0%) in contrast to our expectations. However, there was a relatively high comparable prevalence of mild depression in both study groups (more than 80% of patients in each group). Despite these data, subjectively perceived depression was described in only 24.5% of patients with the DF and 7.3% of diabetic controls. The prevalence of depression in patients with the DF may thus be underestimated. This was reported in a study by Rubin et al, where diagnosed and treated depression was registered only in 25% of patients who needed therapy [32].

We assume that patients with DF had no severe depression because of good mental health and cognitive functions (evaluated by psychological health domain in WHOQOL-BREF test). The lack of severe depression in those patients could also be secondary to a well-functioning social background similar to that of diabetic controls, consistent with the results found in social relationships domain of QoL questionnaire and in the social support questionnaire.

Another factor that could potentially protect patients with DF against severe depression is good adaptability to stress. We expected to find higher psychological pressure in our patients with DF; however, this evaluated parameter was not so markedly expressed in such risk population as in patients after major amputation. Based on completed questionnaires (social readaptation scale), only 31% of patients with DF reported a high stress load with a high risk of somatization compared to 17% of controls. Similar tendencies were shown in the results of the scale of psychological load and stress. We therefore speculate that our patients with DF may have a better ability to adapt themselves to chronically applied stress or have different personal characteristics preventing a negative effect of stress loading. This finding corresponds well to the finding of only a small number of DF patients exhibiting risk behavior, exhibiting hostility, irritability, and frustration.

Suicidal tendencies were found in approximately 5% of patients in both groups. As previously described, it is possible that the chronicity of the condition, the demands placed on patients with respect to their therapy, or the progression to late complications could all lead to suicidal inclinations [33, 34].

Our study provides a comprehensive overview on the psychological characteristics of patients with DF on stress and the patient's socioeconomic profile. This is different from other studies which focused on the possible impact of DF on quality of life [33] or depression [35]. The significant negative effect of DF on the quality of life, even when the social background was relatively good, was a remarkable finding. This comprehensive study is even more valuable since we compared our data with diabetic subjects without serious late complications of the disease with the exception of painless sensory peripheral neuropathy, which in this study had no impact on the outcomes.

There are only a few published studies that have comprehensively compared the psychological issues of patients with DF with diabetic patients without this late complication [31, 36] or with healthy volunteers [33]. However, these published studies had their own limitations in terms of data collection or sample size. To date, social issues have not been extensively studied in patients with DF. There exist data mainly in the context of wound healing or the risk of amputations [6, 24].

Perhaps a limitation of this study could be seen in a smaller number of diabetic controls or the choice of the diagnostic tests and questionnaires specifically the quality of life questionnaire. The SF-36 is the tool frequently used to determine the quality of life in patients with DF [31]; however SF-36 is also designed for the general population. NeuroQoL was developed especially for patients with neuropathy to determine the impact of neuropathy on quality of life [37]. We chose the more general WHOQOL test which was designed to be complex, subjective, to emphasize the relative importance of the different areas of life for individuals, and that had already been specifically adapted to our culture [13].

We conclude that patients with DF in comparison with diabetic patients without late complications have significant abnormalities mainly in social areas of their lives as opposed to psychological ones. The results of this study show that patients with DF have a reduced standard of living in contrast to a well-functioning social background. They had no severe depression, but mild forms of depression were present quite commonly in both groups. Good tolerability of, and adaptation to, chronic stress could play a role in the finding of relatively few psychological abnormalities in this group of patients with DF with the exception of those patients who had had major amputations where higher psychological pressure was described.

The poorer standard of living noted in patients with DF is likely to be secondary to economic problems and the low level of self-support. Based on our data, a properly carried out social and psychological examination may be able to detect early problems which may be amenable to change in this group of patients. For example, psychotherapy focusing on cognitive-behavioral techniques, behavioral family therapy, problem-solving skills, and improved patient motivation may lead to improved health status and self-support and reduce further psychological pressure in patients with DF. Social issues and consequent standard of living are more difficult to influence but could possibly be improved by enhancement of work opportunities or by the creation of special work programs designed specifically for such a chronically ill group.

## Figures and Tables

**Table 1:** Baseline characteristics of the study groups.

Evaluated parameters	Patients with DF (DF group; *n* = 104)	Control group (C group; *n* = 48)	*P* value
Gender—men/women (%)	70.9/29.1	58.3/41.7	NS
Age, mean ± SD (years)	59.1 ± 9.9	61.1 ± 10.7	NS
BMI, mean ± SD (kg·m^−2^)	30.3 ± 5.6	28.9 ± 3.7	NS
Type 1 diabetes mellitus/type 2 (%)	21.2/78.8	18.8/81.2	NS
Diabetes duration, mean ± SD (years)	19.2 ± 9.9	10 ± 7.2	*P* < 0.001
HbA1c, mean ± SD (mmol/mol)*	64 ± 19	60 ± 15	NS
DF duration, mean ± SD (months)	14 ± 16.6	—	—
Patients with severe peripheral diabetic neuropathy (%)	97.1%	27.1%	*P* < 0.001

*HbA1c according to IFCC (normal values 28–40 mmol/mol).

**Table 2:** The comparison of quality of life, depression, and stress readaptation between patients with DF and controls.

Instrument	Evaluated parameters (scale)	DF group (*n* = 104)*	C group (*n* = 48)**	*P* value
WHOQOL-BREF (4 domains)	Physical health domain	12.7 ± 2.8	14.7 ± 2.5	*P* < 0.001^†^
	Psychological health domain	14.3 ± 2.6	15 ± 2.5	NS
	Social relationship domain	14.2 ± 2.9	14.8 ± 2.6	NS
	Environment domain	14.1 ± 2.2	15 ± 1.8	*P* < 0.01^††^

Geriatric depression scale	No depression (% of patients)	15.8	10.4	NS
	Mild form of depression (% of patients)	83.2	89.6	NS
	Severe form of depression (% of patients)	1	0	NS

Social readaptation scale	Scale 1 = score < 150 (% of patients)	69.3	83.3	NS
	Scale 2 = score 150–200 (% of patients)	14.9	8.3	NS
	Scale 3 = score 200–299 (% of patients)	8.9	4.2	NS
	Scale 4 = score > 300 (% of patients)	6.9	4.2	NS

*DF group included patients with the DF; **C group included diabetic controls; ^†^using ANCOVA physical health domain was (mean ± SE) 12.7 ± 0.3 in the DF group versus 14.5 ± 0.4 in the C group, *P* = 0.003; ^††^using ANCOVA environment domain was 14.3 ± 0.2 in the DF group versus 14.6 ± 0.3 in the C group, *P* = 0.437.

**Table 3:** The comparison of selected demographic and social characteristics including self-support between the study groups.

Evaluated parameters		DF group (*n* = 104)*	C group (*n* = 48)**	*P* value
Education level	Basic education/training (% of patients)	30.4	8.4	*P* < 0.001
	Secondary without leaving exam (% of patients)	25.5	16.7	NS
	Secondary with leaving exam (% of patients)	30.4	33.3	NS
	University (% of patients)	13.7	41.7	*P* < 0.001

Employment status	Full-time job (% of patients)	11.7	29.2	*P* < 0.001^†^
	Part-time job (% of patients)	4.9	2.1	NS
	Business (% of patients)	4.9	18.8	*P* < 0.001^†^
	Old age pension (% of patients)	34	39.6	NS
	Disability pension (% of patients)	41.7	6.2	*P* < 0.001^†^
	Others (% of patients)	2.9	4.2	NS

Self-support	Fully self-support (% of patients)	38.1	87.2	*P* < 0.001^††^
	Partially self-support (% of patients)	55.7	12.8	*P* < 0.001^††^
	Fully dependent on the assistance of other persons (% of patients)	6.2	0	NS

*DF group included patients with the DF; **C group included diabetic controls; ^†^using logistic regression analysis *P* = 0.0449 after adjustment for diabetes duration; ^††^using logistic regression analysis *P* < 0.001 after adjustment for diabetes duration.
